# Risk factors for diarrhoea and malnutrition among children under the age of 5 years in the Tigray Region of Northern Ethiopia

**DOI:** 10.1371/journal.pone.0207743

**Published:** 2018-11-26

**Authors:** Araya Gebreyesus Wasihun, Tsehaye Asmelash Dejene, Mekonen Teferi, Javier Marugán, Letemichal Negash, Dejen Yemane, Kevin G. McGuigan

**Affiliations:** 1 College of Health Sciences, Department of Medical Microbiology and Immunology, Mekelle University, Tigray, Ethiopia; 2 College of Health Sciences, Department of Medical Microbiology, Axum University, Tigray, Ethiopia; 3 College of Natural and Computational Sciences, Department of Biology, Mekelle University, Tigray, Ethiopia; 4 Department of Chemical and Environmental Technology, Universidad Rey Juan Carlos, C/ Tulipán s/n, Móstoles, Madrid, Spain; 5 College of Health Sciences, School of Public Health, Department of Environmental Health, Mekelle University, Tigray, Ethiopia; 6 Department of Physiology and Medical Physics, RCSI, Dublin 2, Ireland; Arizona State University, UNITED STATES

## Abstract

**Background:**

Diarrhoea and malnutrition are the leading cause of morbidity and mortality among children in areas with poor access to clean water, improved sanitation, and with low socioeconomic status. This study was designed to determine the prevalence of diarrhoea, malnutrition and risk factors among children aged 6–59 months in the Tigray Region of Northern Ethiopia.

**Methods:**

A community based cross-sectional study design was conducted from June to August 2017 to assess the magnitude and factors associated with diarrhoea and malnutrition among children. A standardized questionnaire was used to collect data on diarrhoea, environmental, demographic and behavioural factors from 610 mother-child pairs. Anthropometric measurements were collected from the children. SPSS ver.21 statistical software was used for analysis. Factors associated with diarrhoea and nutritional status were identified using bivariate and multivariate logistic regression. A p-value ≤ 0.05 was considered statistically significant.

**Results:**

Of the 610 children monitored in this study, the incidence of diarrhoea among 6–59 month-old children in the two weeks preceding the day of the interview day was 27.2% (95% CI: 23.6–31%). Specifically, 35.9%, 9.7%, and 1.8% had 1–2, 3–4 and 5–6 times of diarrhoea episodes in a one year of time, respectively. The prevalence of stunting, underweight, wasting, and acute under-nutrition were 36.1% (95% CI: 31–38.6%), 37% (95% CI: 32–39.6%), 7.9% (95% CI: 5.5–9.7%), and 5.4% (95% CI: 3.8–7.4%), respectively. In a multivariate logistic regression analysis, type of drinking water source [AOR = 3.69; 95% CI: 2.03–6.71], mothers not hand washing at critical times [AOR = 15.42; 95% CI: 2.02–117.78], improper solid waste disposal [AOR = 12.81; 95% CI: 2.50–65.62], and child age (36–47 months) [AOR = 2.57; 95% CI: 1.45–4.55] were found to be predictors of diarrhoea. Being within the age range of 12–23 months was a predictor for wasting [AOR = 4.38; 95% CI: 1.61–11.90] and being underweight [AOR = 4.4; 95% CI: 1.7–11.2]. Similarly, the age range of 36–47 months was associated with wasting [AOR = 2.3; 95% CI: 1.45–3.85] and stunting [AOR = 1.7; 95% CI: 1.03–2.67]. Family size (less than 4) [AOR = 0.56; 95% CI: 0.368–0.959] was inversely associated for wasting.

**Conclusions:**

Our study revealed that the problem of diarrhoea and malnutrition amongst 6–59 months children in the study area was significant. Access to clean water was the main problem in the study area. Hence, improving access to clean water and providing health education to mothers on personal and environmental hygiene, and proper waste disposal could improve diarrhoea in the study area. Intervention on children’s nutrition should also be implemented to minimize the problem of malnutrition.

## Introduction

Diarrhoea and malnutrition cause more morbidity and mortality among children under 5 years old world wide [1]. Among the infectious disease, diarrhoea is the second cause of post-neonatal under 5 year death accounting for 2.5 million each year worldwide, higher than that of AIDS, malaria, and measles combined. This diarrhoeal associated mortality is more concentrated in sub-Saharan African countries (88 per 1000 live births) [2, 3]. Globally, more than 3 million children under the age of 5 years die per year due to malnutrition [4]. Malnourished children are negatively and irreversibly affected in their school performance, physical growth and cognitive development [5].

Malnutrition and diarrhoeal mortality have a bidirectional association [6, 7]. Malnutrition causes immune-deficiency and increased susceptibility to infections such as diarrhoea [8]. Diarrhoea in turn causes malnutrition through reduced food appetite, energy intake, nutrient loss and mal-absorption [9].

Diarrhoea and malnutrition are associated with water, sanitation, and hygiene through different mechanisms. For example, faecal exposure through contaminated water, unimproved sanitation and poor hygiene, leads to diarrhoea which affects physical and mental growth of a child [10–12].

In 2003 the Ethiopian government implemented the Health Extension Program (HEP) as a means of providing comprehensive, equitable and affordable healthcare for the rural population on the basis of promotive, preventive and basic curative services [13]. As a result the 2016 Demographic and Health Survey of Ethiopia (EDHS) report showed an overall decline in the under-five death rate from 166/1000 in 2000 to 67 death/1000 births 2016 [14]. Nevertheless, the national levels of diarrhoea (12%), stunting (38%), wasting (10%), and underweight (24%) reported in the EDHS highlights that malnutrition and diarrhoea still present significant risks for children under five.

In Tigray regional state, the problem of malnutrition is shown by the rates of stunting (39.3%), wasting (11.1%), and underweight (23%), which are all marginally above the national average [14]. Prevalence of diarrhoea among children under 5 years in different regions of Ethiopia ranges from 18 to 31% [15–21]. Similarly, prevalence of stunting (https://doi.org/10.1186/s12889-015-1370-9 24.9–47.6%), wasting (9–13.4%) and being underweight (14.3–29%) has been reported [22–24]. These studies, however, are focused on either diarrhoea or malnutrition alone, and the association of diarrhoea with malnutrition or vice versa has not being deeply studied. Besides, these studies are done in different regions of the country where socio economic, cultural and access to safe water is greatly varied.

There is scant data on prevalence of diarrhoea and malnutrition and risk factors among children aged between six months to fifty nine months in the study area. However, given the poor access to clean water and improved sanitation, low socioeconomic status, and inadequate hygiene, the problem is expected to be high.

Hence, addressing this knowledge gap in the rural community of the study area is reasonable to help policy makers and implementers in order to plan and design proper intervention strategies to prevent diarrhoea and malnutrition associated morbidity and mortality among under five children.

## Materials and methods

### Study design and study population

#### Study setting

Tigray is the northernmost of Ethiopia’s federal states located at 12^o^15’ - 4^o^57’ longitude and 36^o^27’- 39^o^59’ latitude with population size of 6,960,003 within an area of 41,409.95 km^2.^ The capital city of the State of Tigray is Mekelle, which is located 783 km north of Addis Ababa, the capital of Ethiopia. Based on the 2007 Census conducted by the Central Statistical Agency of Ethiopia (CSA), Mekelle has a total population of 215,914 [25].

**Fig 1:**
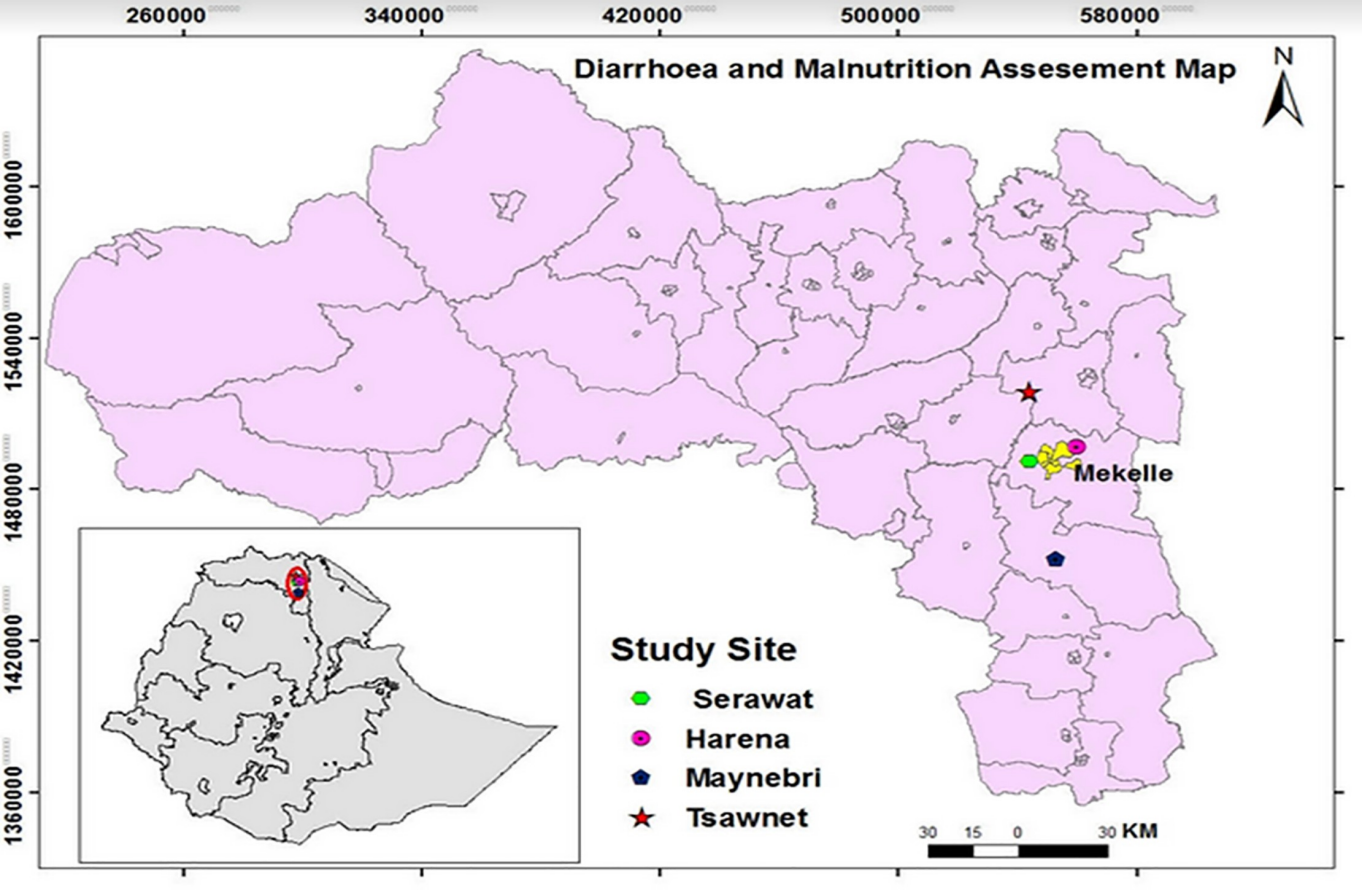
Map of the study area.

This community based cross-sectional study was carried out from June–August 2017 in rural communities surrounding the Mekelle zone, Tigray region, northern Ethiopia. From the total of 32 kebelles (a kebelle is the smallest administrative unit in Ethiopia) found in the surrounding districts, 4 sites were selected using a simple random lottery method. The names of the study sites and their respective GPS are: Serawat 13°29'12.78"N, 39°24'28.10"E, Harena 13°33'0.88"N, 39°32'24.60"E, Maynebri 13°8'12.25"N, 39°29'16.53"E and Tsuwanet 14°0'49.27"N, 39°27'23.39"E. The study area has a semi-arid climate and gets rainfall, mainly from the mid of June to mid of September, and farming is the common means of income among the inhabitants. Likewise, the population typically experience poor sanitation, poor access to safe drinking water, and low socioeconomic status (“**Fig 1”).**

#### Study participants

The source population was all households with children (paired with their mothers) between the ages of 6–59 months in all the kebelles. To select the study participants, we randomly selected 6–59 months old children (paired with their mothers) who were permanently resident in the districts for at least six months.

#### Sample size and sampling technique

The sample size of the study was determined using a single population proportion formula, considering an estimate of 26.1% expected prevalence of diarrhoea among children younger than 5 years old [26]. Assuming any particular outcome to be within a 5% marginal error and a 95% confidence interval of certainty, the final sample size with a design effect of 2 and a 97% response rate was determined to be 610 mothers/children pair.

We used a multistage stratified sampling technique to identify study participants after the kebelles were stratified. Out of 32 kebelles, 4 were selected by lottery method. In these selected kebelles, 2,674 children who are 6–59 months were identified with their respective households using the registration at health posts and though the Health extension workers (HEW).

We allocated the calculated sample proportionally to the selected kebelles based on the total number of households with 6–59 months children in each kebelle, and study participants were then identified using simple random sampling of the households. Accordingly, the distribution of households with respect to the kebelles was, 133 from Tsawnet, 142 from Harena, 158 from Serawat, and 177 from Mynebri. In cases where more than one child between 6–59 months of one mother were in the household, the eldest child was included in the study.

### Variables

#### The primary outcome

Diarrhoea among children 6–59 months in the past 14 days. Secondary outcomes were stunting, wasting and being under-weight.

**Independent variables.** Socio-economic variables: Mother’s and husband’s educational status, family size, family income, sex of child, age of child, number of children 6–59 months.

Environmental and behavioural variables: Use of soap for hand washing, mother’s hand washing at critical times, treatment of drinking water, latrine availability, distance from water source, type of drinking water source, child hand cleanliness, child finger nail status, and method of solid waste disposal of child faeces.

#### Operational definition

- **Hand washing at all 5 critical times:** If a mother/caregiver practiced simple hand washing before eating, before food preparation, before child feeding, after child cleaning and after latrine visiting, this was considered all practiced, if all five action were done. If not, it was considered as "partially practiced".
- **Proper solid waste disposal:** is a way of disposal of ordinary domestic waste which includes burning, buried in pit or stored in a container, compost, and whereas disposing in open field was considered as improper disposal.
- **Unimproved water source:** when the people use water for drinking from a river, pond, well or unprotected spring.

### Data collection

Data on socioeconomic, environmental, behavioural factors and health related were collected using a structured questionnaire (translated from English and printed in the local Tigrigna language). The data were collected using a face-to-face administrated questionnaire and an observation method by trained data collectors, under the supervision of the investigators. To assess the childhood illness, the mothers were asked whether their children had been affected by diarrhoea in the past two weeks. In addition to this, mothers were also asked the number of diarrhoeal episodes experienced by their children in the last year. Diarrhoea was defined as having three or more loose or watery stool in a 24-hour’s period within the two week period prior to the survey [27]. The collected data were checked for errors and incompleteness on a daily basis. Finally, the data were coded, entered and analyzed using SPSS software, version 21 (Chicago, IL, USA).

## Anthropometric measurement

Height/length and weight measurement of the child were taken by the health extension workers who are already trained by the government and work the health posts. Digital weight scale was used to measure weight of children. Weight was recorded to the nearest 0.1 kg with the child barefoot and wearing light clothing. Children who were unable to stand on the scale, and 6–24 months were weighed with the mother or legal guardian, then the mother/ guardian was weighed alone, and the differences were used to obtain the net weight of the children.

The height of the children was measured using a calibrated height measuring board. A child who could not stand erect was measured in supine position. A child who could stand erect and above 24 month was measured standing against a calibrated height measuring board. The height measurement was taken to the nearest 0.1 cm. Mid-Upper Arm Circumference (MUAC) was measured at the mid-point between the tip of the shoulder and the tip of the elbow of the left upper arm using non-stretchable tape to the nearest 1 mm. MUAC below 12.5 cm was considered as indicator of an acute under nutrition.

We used WHO Anthro version 3.2.2 software to convert the anthropometric measures; age, weight, height/length values into Z-scores of the indices; Weight-for-Height (WHZ), Weight-for-Age (WAZ), and Height-for-Age (HAZ) taking gender of the child into consideration using WHO 2006 standards [28]. In all analyses Z-scores <-6.00 or >6.00 were considered outliers and excluded from the study. Accordingly, HAZ (n = 13), WAZ (n = 1) and WHZ (n = 1) were excluded from the study.

### Statistical analysis

Frequency distributions of socio-demographical and behavioural characteristics of participants were explored. Continuous variables were expressed as mean ± standard error of mean; whereas, categorical variables were expressed as number (percentage, %). Chi-square tests were used to evaluate the differences in the distribution of categorical variables for study groups. Children who were undernourished with a Z-score value less than or equal to −2 S.D. were characterized as stunted (low height/length for-age) (HAZ), wasted (low weight-for-height) (WHZ), and underweight (low Weight for-age) (WAZ), respectively.

Bivariate logistic regression analysis was done to identify factors associated to diarrhoea and under- nutrition. Factors associated with the outcome variables (diarrhoea and under- nutrition) in bivariate analysis at a significance level of 0.25 were identified and exported to multivariable logistic regression analysis. Confidence intervals (CI) of 95% were reported for each odds ratio (OR). All reported p-values were two-tailed, and statistical association was set significant at p-value ≤ 0.05.

## Ethical considerations

Ethical clearance was obtained from Mekelle University; College of Health Science Institutional Review Board (IRB) (**ERC 0844/2016**). The Tigray Regional Health Bureau was an active collaborator in the research project. Written consent was obtained from each mother / guardian of each child. Participants were asked to enroll and were told that they had the right not to respond to questions that they did not want to and could stop at any point in the survey if they wanted. Children with any diarrhoea and acute malnutrition who did not visit health centres during the study period were linked to the nearby health centres by the clinical nurses who were data collectors. Treatment costs for all the diarrhoeic children were covered from the project fund.

## Results

### Socio-economic and demographic data of the study households

A total of 610 under-five children/mothers pair participated in the study with a 100% response rate. The majority, 598 (98%) of the interview respondents comprised mothers of the 6–59 months children and the remaining 12(2%) were care-givers older than 18 years. The majority of the mothers (90.4%) were married. Most mothers (79%) had one under-five child. According to the results of the study, 58% mothers and 44.9% of the fathers were illiterate (unable to read or write). Regarding employment status of the mothers and husbands, 92.1% mothers were housewives and 75.6% of the fathers were farmers. The majority of households (76.4%) had more than four members. The majority of households (73.9%) had a family monthly income between 500–2000 Ethiopian Birr ($20–80 USD, **Table 1**).

**Table 1:** Socio-demographic characteristics of mothers with children 6–59 months, in Enderta district, Tigray region, northern Ethiopia, June-August, 2017.

Variables	Frequency	%
**Marital status of the mother**		
Married	551	90.4
Single	2	0.3
Divorced	57	9.3
**Number of children under 5 years of age**		
<2	482	79
≥2	128	21
**Number of family members**		
<4	144	23.6
_≥_4	466	76.4
**Religion of the parents**		
Orthodox Christian	595	97.5
Muslim	13	2.1
Catholic	2	0.3
**Educational level of the mother**		
Illiterate	354	58.0
Primary school graduate	193	31.6
Secondary school graduate	54	8.9
Diploma and above	9	1.5
**Occupation of the mother**		
House wife	562	92.1
Governmental employee	19	3.1
Self employed	29	4.8
**Education of husband (N = 551)**		
Illiterate	274	44.9
Primary school complete	212	34.8
Secondary school complete	51	8.4
Diploma and above	14	2.3
**Occupation of the husband(N = 551)**		
Farmer	461	75.6
Governmental employed	20	3.3
Self employed	70	11.5
Family monthly income [Ethiopia birr[*		
<500	99	16.2
500–2000	451	73.9
>2000	60	9.8

*1birr = 0.043 US dollar

### Environmental health conditions the study households

As to the hand washing habit of mothers at critical times, the majority 93.9% were partially washing at all time. More than half, (60.2%) of the mothers used soap for hand washing. Access to improved waster was only reported among 23% of the households. Regarding proximity of house to main water source, 32% spent 30 minutes, and 33.2% of them travelled a round-trip from 60–120 minutes to fetch water. Household water treatment (any means) was practiced among 150 (24.6%) households. Regarding the latrine availability, 59.7% of the households had their own latrine facilities (**Table 2)**.

**Table 2:** Environmental characteristics of households in Tigray region, Northern Ethiopia (June-August, 2017).

Variables	Frequency	%
**Mother's hand washing at all 5 critical times**		
All practiced	37	6.1
Partially practiced	573	93.9
**Cleaning materials used to wash hands**		
Water only	243	39.8
Water and soap	367	60.2
**Habit of eating vegetable and fruits (N = 578)**		
Never	106	17.4
Some times	472	77.4
Always	32	5.2
**Source of drinking water of the family**		
Protected	140	23
Unprotected	470	77
**Time taken to fetch water(in minutes)**		
<30	195	32.0
30–59	213	34.9
60–120	202	33.2
**Use of household water treatment**		
Yes	150	24.6
No	460	75.4
**Means of in-house water treatment used (N = 150)**		
Boiling	69	11.3
Chlorination	73	12.0
Filtration	8	1.3
**Habit of mother washing with soap before child feeding**		
Never	80	13.1
Some times	344	56.4
Always	186	30.5
**Presence of domestic animals in house**		
Yes	486	79.7
No	124	20.3
**Latrine availability**		
Pit latrine with slab	161	26.4
Pit latrine without slab (open pit)	203	33.3
Open defecations	246	40.3
**Solid waste disposal**		
Proper	317	52
Improper	293	48

#### Demographic and health characteristics of the children

The results of the study showed that 53.4% of the children were male, and the mean age of the children was 35.2 months with S.D. ±14.7 months. Regarding gastro-intestinal symptoms during the data collection, 22% had some form of GI symptoms, with diarrhoea being the most common. The point prevalence for diarrhoea was 13.1%. Two hundred and nineteen, (35.9%) of the children had diarrhoea episodes 1–2 times in the previous one year period. Diarrhoea prevalence among children in the two weeks preceding the interview day was 166 (27.2%) (**Table 3**).

**Table 3:** Health and demographic related Characteristics of 6–59 months children in Tigray region, Northern Ethiopia (June- August, 2017).

Variables	Frequency	%
**Gender of the child**		
Male	326	53.4
Female	284	46.6
**Age of the infant (months)**		
6–11	32	5.2
12–23	108	17.7
24–35	160	26.2
36–47	149	24.4
48–59	161	26.4
**Habit of eating vegetable and fruits (N = 578)**		
Never	93	16.1
Some times	454	78.6
Always	31	5.3
**Habit of child playing in soil (N = 578)**		
Never	10	1.7
Some times	127	22
Regularly	441	76.3
**Finger Nail status of the child**		
Trimmed	315	51.6
Untrimmed	295	48.4
**Child hand cleanness**		
Clean	367	60.2
Unclean	243	39.8
**Current GI symptom (N = 134)**		
Yes	134	22
No	476	78
**Type of GI (N = 134)**		
Abdominal pain	24	3.9
Diarrhoea	80	13.1
Fever	2	.3
Vomiting	2	.3
Diarrhoea and abdominal pain	26	4.3
**Diarrhoea episode in last two weeks**		
Yes	166	27.2
No	444	72.8
**Diarrhoea episode of child this year(N = 578)**		
1–2	213	36.9
3–4	56	9.7
5–6	10	1.7
No	299	51.7
**Presence of skin diseases**		
**Yes**	174	29.5
**No**	436	71.5
**Type of skin disease (N = 174)**		
Scabies	22	3.6
T. capitis	125	20.5
Scabies and T. capitis	3	.5
Others (Cellulitis +Folliculitis)	24	3.9
No	436	71.4

#### Prevalence of malnutrition

**Fig 2:**
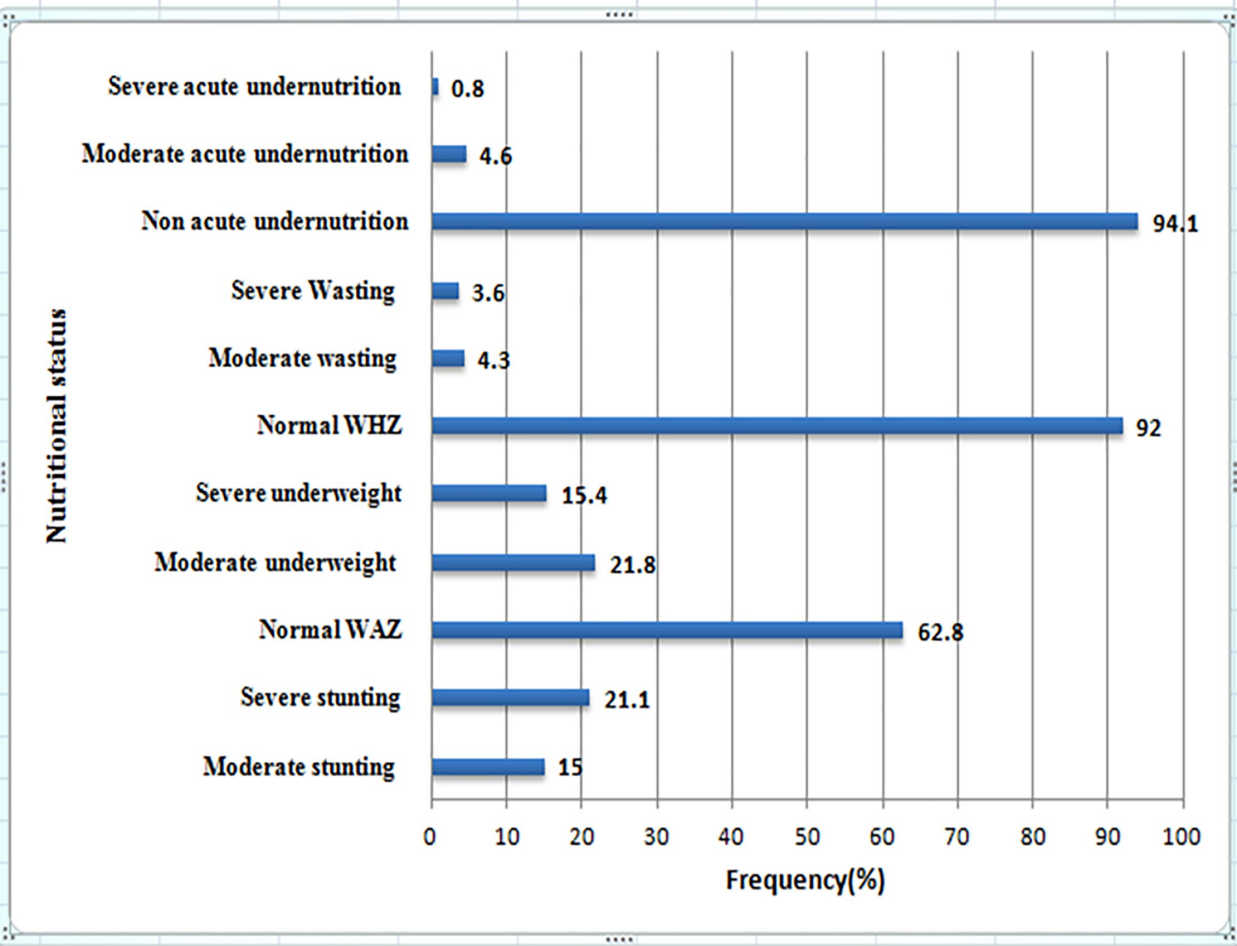
Proportion of malnutrition among children aged 6–59 months in Tigray region, Northern Ethiopia, (June-August, 2017).

Regarding nutritional the status of the children, stunting, underweight, and wasting were seen in 36.1%, 37% and 7.9%, respectively. Prevalence of severe stunting, underweight and wasting among the children were 21.1%, 15.4% and 3.6%, respectively. Middle upper arm circumstance (MUAC) measurement indicated that 5.4% of the children were undernourished (<12.5 cm) (“**Fig 2”).**

Adjusting for other confounding variables, children whose mothers didn’t wash their hands at all the critical times were 15.4 times [AOR = 15.4; 95% CI = 2.02, 117.78] more likely to have diarrhoea compared to those whose mothers did wash at these times. Children who drank from unimproved sources of water were 3.69 times [AOR = 3.69, 95% CI = 2.03, 6.72] more likely to have diarrhoea compared to those using improved water sources. The odds of a child contracting diarrhoea were 12.8 times higher [AOR = 12.81; 95% CI = 2.50, 65.62] among the households who disposed of their solid waste improperly comparing those who dispose properly.

Children who played regularly in soil [AOR = 1.66; 95% CI = 0. 953, 2.881], had untrimmed finger nail [AOR = 1.576; 95% CI = .598, 4.15], 6-11months age [AOR = 1.5; 95% CI = 0.48, 4.40], who had unclean hands [AOR = 1.2; 95% CI = .71, 1.9] were 1.7, 1.6, 1.5, and 1.2 times more likely to contract diarrhoea, respectively. However, these differences were not statistically significant (**Table 4).**

**Table 4:** Multivariate logistic regression analysis of diarrhoeal disease with selected predictor variables among 6–59 months children in Tigray region, Northern Ethiopia (June- August 2017).

Predictor Variables	Diarrhoea [N = 166)		COR (95%CI)	P-value	AOR (95%CI)	P -value
	**Yes**	**No**				
**Mother hand washing at all critical times**						
All practiced	1	36	Ref.		Ref.	
Partially practiced	165	408	14.6[1.98, 107.06 ]	0.009**	**15.42[2.02, 117.8]**	0.008**
**Source of drinking water of the family**						
Improved	17	123	Ref.		Ref.	
Unimproved	149	321	3.36[1.95, 5.78 ]	0.000**	3.69 [2.030, 6.7]**	.000**
**Mother’s washing with soap before child feeding**						
Always	36	150	Ref.		Ref	
Some times	113	231	1.83[0.361, 0.94]	0.027**	2.04[1.63, 2.56]	0.030**
Never	17	63	2.36[0.35, 0.2.57]	.013**	3.706[2.67, 6.3]	0.000**
**Child hand cleanness**						
Clean	86	281	Ref.		Ref.	
Unclean	80	163	1.604[1.12, 2.300]	0.010**	1.16[.71, 1.89 ]	0.56
**Habit of child soil playing [n = 578]**						
Never	10	38	Ref.		Ref.	
Some times	113	231	0.552[0.31, 0.99]	0.045**	1.336[.378, 4.73]	.653
Regularly	35	150	1.12 [.588, 2.15]	0.123	1.66[.953, 2.88 ]	0.072
**Solid waste disposal**						
Proper	22	29	Ref.		Ref.	
Improper	144	415	2.19[1.22, 3.93]	0.009**	**12.81[2.50, 65.62]**	0.002**
**Latrine presence**						
Yes	96	268	Ref.		Ref.	
No	70	176	1.12[.78, 1.61]	0.556	1.02 [0.39, 2.72]	0.962
**Finger nail status**						
Trimmed	76	239	Ref.		Ref.	
Untrimmed	90	205	1.38 [.97, 1.98]	0.077	1.576[.598, 4.15 ]	0.358
**Distance from water source**						
<30	45	150	Ref.		Ref.	
30–59	60	153	0.765[.489, 1.20]	0.241	1.279[.364, 4.491]	0.701
60–120	61	141	0.69 [.443, 1.08 ]	0.110	1.079[.280, 4.155]	0.912
**Child age(Months)**						
6–11	6	26	2.31 [.90, 5.947]	0.082	1.46[.48, 4.40]	0.507
12–23	38	70	0.982[.589, 1.638]	0.946	0.611[.342, 1.09]	0.095
24–35	41	119	1.55[.96, 2.50 ]	0.075	1.26 [.751, 2.13]	0.377
36–47	25	124	2.65[1.54, 4.53 ]	0.00**	**2.57[1.45, 4.55 ]**	0.001**
48–59	56	105	Ref.		Ref.	
**Household water treatment**						
Yes	46	104	0.798[.53, 1.20]	0.274	0.997[.63, 1.6]	0.997
No	120	340	Ref.		Ref.	

** = statistically significant

#### Factors associated with diarrhoea and malnutrition among 6–59 months children

From **Table 5** we can see that, children from a household who have ≥4 family members were 1.4 times more likely [AOR = 1.4; 95% CI = 0.94, 2.2] to be under-weight compared to families with <4 members. However, the differences of mother’s employment and family size were not statistically significant. Children whose family members numbered less than 4 were 60% less likely to be wasted compared those who were ≥ 4 [AOR = 0.60; 95% CI = .368, 0.959]. Children whose age was from 36–47 months had 1.7 times more likely [AOR = 1.66; 95% CI = 1.03, 2.67] to be stunted compared to those in the 48–59 months age which was statistically significant (p = 0.037). Likewise, children at the age of 12–23 months [AOR = 4.4; 95% CI = 1.61, 11.90] and 36–47 months [AOR = 2.4; 95% CI = 1.45, 3.85] were 4.4 and 2.4 times more wasted, respectively compared to the children at the age of 48–59 months (**Table 5*)*.**

**Table 5:** Multivariate logistic analysis of malnutrition with selected predictor variables among 6–59 months in Tigray region, northern Ethiopia, June-August, 2017.

Predictor variables	Stunting			COR(95%CI)	P-value	AOR(95%CI)	P-value
	Yes		No				
**Mother's hand washing at all critical times**							
All practiced	16		21	Ref.		Ref.	
Partially practiced	192		368	192	368	1.46[.75, 2.86 ]	0.27
**Cleaning materials used to wash hands**							
Water only	76		163	Ref.		Ref.	
Water and soap	132		226	132	226	.0798[.56, 1.43]	0.203
**Source of drinking water of the family**							
Improved	54		83	Ref.		Ref.	
Unimproved	150		306	1.29 [.87, 1.92]	.201	1.15[.80, 1.65]	.449
**Latrine presence**							
Yes	132		224	Ref.		Ref.	
No	76		165	1.92 [.877, 4.21]	0.103	1.78 [.81, 3.94]	.152
**Age of the infant (Months)**							
6–11	14		17	1.92 [.877, 4.21]	0.103	1.78 [.81, 3.94]	.152
12–23	35		69	1.2 [.697, 20.1]	0.532	1.15 [.675, 1.96 ]	.605
24–35	49		105	1.09[.68, 1.76]	0.727	1.10[.68, 1.78]	.704
36–47	62		86	1.68 [1.05, 2.7]	0.03	1.66[1.03, 2.67]**	.037**
48–59	48		112	Ref.		Ref.	
**Underweight**							
**Number of family**							
<4	45		99	.73 [.48, 1.06]	0.097	**0.59[.368, 0.96]**	.033**
_≥_4	181		284	Ref.		Ref.	
**Occupation of the mother**							
House wife	215		346	Ref.		Ref.	
governmental employee	7		15	0.43[.14, 1.31]	.137	.494[0.16, 1.54 ]	.225
Self employed	4		22	0.51 [.215, 1.22]	0.13	.542[.22, 1.34 ]	.186
**Mother’s hand washing at all 5 critical times**							
All practiced	8		29	Ref.		Ref.	
Partially practiced	218		354	2.2 [1.00, 4.97 ]	0.049	**1.96[.85, 4.51**]	0.114
**Age of the infant**							
6–11	13		19	Ref.		Ref.	
12–23	49		58	1.235[.55, 2.75 ]	0.606	1.23 [.54, 2.79 ]	0.618
24–35	49		111	.645[.295, 1.41]	0.272	.57[.26, 1.27]	0.167
36–47	71		78	1.33 [.61, 2.89]	0.47	1.13[.51, 2.49	0.771
48–59	44		117	.55[0.25, 1.21]	0.136	.46 [.21, 1.03 ]	0.059
**Wasting**							
**Age of the infant**							
6–11	2		30	1.47[0.29, 7.41]	0.643	1.2 [0.21, 6.76]	.838
12–23	18		90	4.4 [1.77, 10.9 ]	.001	4.38[1.61, 11.9]**	.002**
24–35	11		148	1.64[0.62, 4.33]	0.322	1.74 [.62, 4.89 ]	.29
36–47	10		139	2.4[1.51, 3.9]	0.000	2.3 [1.45, 3.85 **]**	.001**
48–59	7		154	Ref.		Ref.	
**Educational level of the mother**							
Illiterates	26		327	4.9 [2.1, 5.59 ]	0.289	4.3[1.46, 2.142]	0.318
Primary school completed	18		175	3.4 [1.45, 6.68 ]	0.25	3.4 [1.09, 5.37]	0.29
Secondary school complete	4		50	1.54[1.59, 7.04]	0.09	1.21 [1.20, 6.09]	0.106
Diploma and above	0		9	Ref		Ref	

** = statistically significant

## Discussion

This study investigated prevalence of diarrhoea and malnutrition among children in the 6 month -59 month age range in a rural community which is an important public health concern. Overall, the prevalence of diarrhoea, stunting, underweight, and wasting were 27.2%, 36.1%, 37%, and 7.9%, respectively. Diarrhoea prevalence 166/610 (27.2%) in the study area was in line with the findings of previous studies in Nekemte town, western Ethiopia 28.9% [20], Jigjiga District, eastern Ethiopia 27.3% [29], northeast Ethiopia 26.1% [26], Sheko district, southwest Ethiopia 25.5% [21], and Kashmir, India 25.2% [30].

However, the current finding was approximately 3 times higher than the finding of the 2016 EDHS, in which the magnitude of diarrhoeal disease among children younger than 5 years old was 12% [14]. It was also higher than the findings of similar studies conducted in other parts of Ethiopia such as, Shebedino district, southern Ethiopia 19.6% [31], Bahir Dar, northwest Ethiopia 24.9% [32], Mecha district, northwest Ethiopia 18.0% [15] Jabithennan District, northwest Ethiopia 21.5% [19], Sebeta town, southwest Ethiopia 9.9% [33], Harer, eastern Ethiopia 22.5% [17], Sheko district, southwest Ethiopia 6.4% [21], and Rusizi district, Rwanda 8.7% [34]. Our result was lower than two studies conducted in Arba Minch district, south Ethiopia 31.0% [16] and 30.5% [35], Uttar Pradesh, India 55.6% [36], and northwest Burundi 32.6% [37]. The difference could be attributed to the sample size, seasonal variation in data collection, environmental factors, socio-economic and cultural differences of the study participants.

The higher prevalence of diarrhoea in this study could be due to the fact that our data collection time was from June-August which is rainy season in Ethiopia, whilst the others [17,19,21,29] were collected in the dry season. Seasonal variation has been associated with the occurrence of diarrhoea [36, 38, 39], with higher diarrhoea prevalence during rainy season than dry season [40]. This is due to the contamination of water sources such as rivers, streams, and wells by flood with human excreta from open defecation which is the main risk factor for diarrhoeal disease, especially for children who routinely play in the unhygienic environment.

Similarly, the higher prevalence of diarrhoea than that of Rwanda 8.7% [34], could be due to the fact that the duration for diarrhoea in Rwanda was within 7 days preceding data collection; whereas in our case it was 14 days. Hence the longer time in our case could have increased the chance to get more diarrhoeic children than the 7 days. Other possible reason for our result to be higher than other similar studies [17,19, 21,32, 31–33, 41] is that we included children from 6–59 months while they included all under five children including 0–6 months. This could have its impact in the diarrhoea prevalence since children below six months are exclusively breastfed and don’t have exposure to the external environment, therefore, their odds of having diarrhoea is lower than those who have started complementary food, which in turn decreases the diarrhoeal prevalence [35,42].

Source of drinking water was independently associated with the occurrence of child hood diarrhoea. Children who drink from unimproved source of water were 3.7 times [AOR = 15.419, 95%CI = 2.02, 117.78] more likely to have diarrhoea compared to those who use improved water sources. This was similar to the study of Abdiwahab et al. [29]. The wider confidence interval is because the number of households who have accesses to improved water source were few (23%) compared to those who don’t have (77%).

Poor maternal hand washing practice without soap before child feeding was positively associated with the occurrence of diarrhoeal morbidity among children. Children whose mothers always wash their hand using soap before child feeding had 56% [AOR = 0.56; 95% CI = 0.33, 0.93] lower risk of becoming diarrhoeic compared those who don’t wash. This was supported by other similar studies that proper hand washing before feeding children plays a great role in the prevention of diarrhoea and other diseases [30, 35, 43–46].

In addition, mothers’ habit of hand washing at all critical times showed a remarkable difference with childhood diarrhoea in this study. Only 6.1% of the interview mothers replied that they regularly wash their hands at all the five critical times. As a result, the odds of diarrhoea among children whose mothers didn’t wash at all the critical times was 15 times [AOR = 15.4; 95% CI = 2.02, 117.78] higher compared to children whose mothers practiced hand washing at critical time with soap. This was in agreement with similar studies conducted elsewhere [21, 47–50].

We have also found that improper solid waste disposal is an independent predictor for diarrhoea in the study area. The odds of a child contracting diarrhoea was 12 times higher [AOR = 12.81; 95% CI = 2.50, 65.62] among the households who dispose of their solid waste improperly compared to those who dispose properly. This result was consistent with other reports [21, 47, 48, 51], where poor waste disposal is the environmental factor most often linked to diarrhoea. Poor waste disposal is attributed to direct contact with human excreta when the child starts to crawl, and easily accessible for vector and rodents, which are means of diarrhoea transmission.

In this study, children whose mothers had no formal education had higher incidence of diarrhoea compared with those children whose mothers were educated. However, in a multivariate analysis it was not statistically significant. This contrasts with previous studies who reported significant association between diarrhoea and education status of the mother [21, 26, 29, 35,52].

Surprisingly, household water treatment (boiling and chlorination were the most commonly used in the study area) was not found to be statistically significant in diarrhoea prevention among children in the study area. This is in contrast with studies from Rwanda [34] and Burundi [37]. This could be due to the storage condition and hygienic status of the storage material that creates a risk of recontamination of water after treating. We also observed that mothers complained about the change in water taste after chlorination and children preferred to drink the un-chlorinated rather than chlorinated water. This highlights the need for other household point of care for water treatment like solar disinfection (SODIS) which have shown promising results in child hood diarrhoea reduction in countries such as South Africa [53], Cambodia [54], and southern India [55].

The odds of diarrhoea in children aged 36–47 months [AOR = 2.57; 95% CI: 1.45–4.55] was 2.57 times more compared to children aged 48–59 months which was similar to previous studies [43, 44]. Studies in Thailand reported children aged 6–23 months were at a higher risk of developing diarrhoea [42, 35]. Other researchers [17, 26, 34, 35, 41, 56] have reported more diarrhoea among children in the age groups 6–11 and 12–23 months. This variation of diarrhoea with age of the infants could be explained by fact that children in the age group of 6–12 start complementary food, have immature immunity, start crawling and have high risk to ingest contaminated materials which may put them at higher risk for diarrhoea [35, 42]. The higher diarrhoea prevalence in children 36–47 months in our study could be due to their higher exposure to external environment, play in unhygienic places, eating by themselves with unclean hands, starting to clean themselves after defecation all of which put them at a higher risk for diarrhoea [43,44].

Family size and child sex [19, 38], number of under five children [17, 26], family monthly income [21], latrine availability [16, 29, 57, 58] were reported as risk factors for the occurrence of diarrhoea by other investigators, but we found no evidence of this.

Nutritional status in this study showed that prevalence of child stunting (36.1%) and wasting (7.9%) were slightly lower in the study area in comparison with the regional prevalence reported by Ethiopian DHS 2016 of 39.3% stunting and 11.1% of wasting. On the contrary, prevalence of underweight (37%) in the study area was very high compared to the regional 23% Ethiopian DHS report [14]. The difference might be due to our small sample size compared to the national data, and the fact that we excluded children under the age of six months. Our stunting prevalence was similar to the study conducted among a pastoralist community of Ethiopia 34.4% [59]. However, it was lower than reported from Bule Hora district, south Ethiopia 47.6% [23]. Variations in nutritional status of children could be the result of socioeconomic, feeding habit of child, environmental hygiene, and cultural difference among societies.

Though it was not statistically significant children whose mothers were either illiterate, or who had only completed elementary school were 4.3 and 3.4 times, respectively, more likely to have wasting than those whose mother were diploma holders. Similarly, stunting was 1.5 times more prevalent among children from mothers who do not have formal education than those who are diploma holders. A statistically significant association has been reported in previous studies between maternal education status with stunting, wasting [60,61], and underweight [59].

High energy expenditure, lower appetite, nutrient losses and mal-absorption caused by diarrheal infections are associated with malnutrition [7]. In this study, however, presence of diarrhoea in the last two weeks prior to data collection was not statistically significant with stunting, underweight and stunting, which contrasts other studies [59,62,63]. This indicates other factors may lead to malnutrition such as shortage of food, limited access to balanced diet both for the mother and the child, and child feeding practices.

Family size (less than 4) [adjusted OR = 0.56; 95% CI: 0.368–0.959] was inversely associated with wasting. This was in line with the report of Ethiopian DHS 2016 which shows that there is an inverse relationship between the length of the preceding birth interval and the proportion of children who are wasted [14]. The longer the interval, the less likely it is that the child will be wasted [59, 64]. One possible reason might be due to the fact that families with more children face more economic problem for food consumption therefore more likely to suffer from poor nutritional status.

Being between the ages of 12–23 months was a predictors for wasting [AOR = 4.38; 95% CI:1.61–11.90], and being underweight [AOR = 4.4; 95% CI:1.7–11.2], respectively. Similarly, 36–47 months [AOR = 1.7;95% CI: 1.03–2.67] was associated with stunting. Sex, monthly income, source of drinking water, maternal occupation, and latrine presence were not associated with malnutrition in the study area.

### Limitations of the study

As the occurrence of diarrhoea was determined according to the mothers/guardians self-reporting without the confirmation of doctor, the study might be affected by social desirability bias. However, to alleviate this problem, we recruited female health extension workers who are part of the community and who have strong relations with mothers to convince mothers to provide the actual information during data collection. Another limitation was since mothers were also asked the number of diarrhoea episodes of their children in the last one year, there could be a recall bias. Again, due to the nature of the study design, cross-sectional, it was not possible to make any interpretation on causal relationship among variables. Lastly, we did not collect data on mother’s size and weight before pregnancy, the birth weight of the children, and the daily caloric intake which could have helped in interpreting the nutritional results of the children.

### Conclusions

The study revealed that more than one fourth of the children in the rural community were diarrhoeic. Access to clean water, safe disposal of solid waste and mother’s hand washing were found to be a risk factor for the diarrhoea in the study areas. Hence regional government and other stake holders should address the availability of safe drinking water and health education on hygiene in the rural community. Our results also highlight the need for more investment and commitment to minimize the magnitude of childhood diarrhoea by designing and implementing prevention strategies, such as mothers’ education on personal and environmental hygiene by integrating with the existing national health extension program. Similarly, prevalence of stunting, underweight, and wasting were high among the children. Therefore, this study underlines the need for an interventions focusing on improving promotion of nutrition, education, and family planning.

## Supporting information

S1 Table Questionnaire. (DOCX)
