# Supplementary material for: Risk factors for diarrhoea and malnutrition among children under the age of 5 years in the Tigray Region of Northern Ethiopia
Source: PLoS One. 2018 Nov 26;13(11):e0207743. doi: 10.1371/journal.pone.0207743 (PMC6257922; doi:10.1371/journal.pone.0207743)
Supplement: S1 Table — (DOCX) [file pone.0207743.s001.docx]

**Annex I: Questionnaire on Socioeconomic and associated risk** factors

Name of the data collector: ___________Date:________, Site/ Kebelle ___________________

**Full name of child _______________________________; Code No. _______**

| Sr. No. | Questions | Responses |
| --- | --- | --- |
| Q 1. | Gender of the child | 1. Male  2. Female |
| Q 2. | Age (months of the child) | 1. 12-23 2. 24-35 3. 36-47 4. 48-59 5. 60-72 |
| Q 3 | Number of family in the house | 1. < 5  2. 5  3. 6  4. >6 |
| Q4 | No of Siblings | ------------ |
| Q5 | Number of under five children | ------------ |
| Q8 | Religion of parents/caretakers | 1. Orthodox  2. Muslim  3. Catholic , 4 Other (specify)_____ |
| Q9 | Educational level of mother/caretaker | 1. Illiterate  2. Primary school  3. Secondary school  4. Diploma and above |
| Q10 | Occupation of the mother/caretaker | 1. Housewife  2. Government employee  3. Private gainful work  4. Farmer  5. Merchant |
| Q11 | Educational level of the father | 1. Illiterate  2. Primary school  3. Secondary school  4. Diploma and above |
| Q12 | Occupation of the father | 1. Government employee  2. Self employed  3. Merchant  4. Farmer |
| Q13 | Family monthly income in birr | 1. < 500  2. 500  3. 2000  4. >2000 |
| Q14 | Do you wash your hand with soap after toilet | 1. Never  2. Sometimes  3. Always |
| Q15 | Habit of eating raw fruits and vegetables of the child | 1. Never  2. Sometimes,  3. Regularly |
| Q16 | Habit of eating uncooked meat of the child | 1. Never  2. Sometimes  3. Regularly |
| Q17 | Source of drinking water for the child | 1. Pipe water,  2. Protected Well,  3. Unprotected well ,  4. Protected spring,  5. Unprotected spring,  6. River/ Pond |
| Q18 | Is there any means of house hold water treatment | 1. Yes  2. No if **No** skip to Q20 |
| Q19 | If yes for Q 18 which one do you use? | 1. Boiling  2. SODIS  3. Chlorine solution  4. Strain it through a cloth  5. Water filter(Ceramic, sand, |
| Q20 | Use of soap for hand washing for the child | 1. Never,  2. Sometimes,  3. Always |
| Q21 | Habit of child playing in soil | 1. Never **if Never skip to Q24**  2. Sometimes  3. Regularly |
| Q22 | Washing hands of child after playing with soil | 1. Never,  2. Sometimes  3. Always |
|  | Hand washing habit before meal | 1. Never,  2. Sometimes  3. Always |
| Q23 | Finger nail status of the child (**Observation**) | 1. Trimmed,  2. untrimmed |
| Q24 | Shoe wearing habit of the child | 1. Never,  2. Sometimes,  3. Regularly |
| Q25 | Do you have domestic animals | 1. Yes **If No skip to Q27**  2. No |
| Q26 | Child Contact with domestic animals | 1. Never  2. Sometimes  3. Regularly |
| Q27 | Do you have a latrine? | 1. Yes  2. No **If No skip to Q 29** |
| Q28 | If **Yes** for Q 27 what type | 1. Yard toilet,  2. Pit latrine with slab  3. Open pit  3. Ventilated Improved Pit latrine |
| Q29 | If the family has no latrine, where do you defecate | 1. Open field  2. From neighbor hood latrine  3. Other (specify)----------------- |
| Q30 | Current symptoms of the child for GI? | 1. Yes 2. No |
| Q31 | If **yes**  for Q 31 which one | 1. Abdominal pain  2. Diarrhoea,  3. Fever,  4. Vomiting  5. Other specify ______ |
| Q33 | Do your child have diarrhea currently? | 1. Yes  2. No if No skip to Q37 |
| Q34 | If **Yes** for Q 33 For how long the diarrhea last? | 1. Less than or equal to 7 days  2. Greater than 7 days |
| Q35 | Number of diarrhea/day | 1. Three times  2. More than three times  3.. Don't know |
| Q36 | Type of diarrhea that the child has | 1. Watery  2. Bloody and mucus  3. Other specify _______ |
| Q37 | Did the child take medication for diarrhea? | 1. Yes  2. No |
| Q38 | If No for Q37, Why? | 1. High cost to buy  2. Distant health center  3. It will cure by it self  4. Other specify________ |
| Q39 | Has the child treated for any IP in the last 1 month? | 1. Yes  2. No |
| Q40 | Was there any de-worming program this year? | 1. Yes  2. No |
| Q 41 | Did your child get Iron supplement? | 1. Yes  2. No |
| Q42 | If No why? | 1. Was not indeed of it  2. Shortage of supply  3. Other specify |
| Q43 | Skin disease | 1.Yes  2. No |
| Q44 | If **Yes** specify | 1. Scabies  2. Fungal(ring worm)  3. Annul( Pin worm)  4. Ulcer  5. Sore  6. Irritation  7. Other specify ______ |
| Q45 | How many time does your child had diarrhea this year? | 1. 1-2  2. 3-4  3. 5-6  4. >6  5. None |
| Q46 | Anemia status( Hg level=________g/L) | 1. Sever Anaemic  2. Moderate Anaemic  3. Mild Anaemic  4. Non Anaemic |
| Q47 | Malnutrition status | 1. Underweight 2. Stunted 3. Wasted |
| Q48 | Parasite isolated from child | 1. *G. lablia* 2. *H. nana,* 3. *S. mansoni* 4. *A. lumbricoides* 5. *T.trichiura* 6. *E. vermicularis* 7. Hookworm 8. *E. histylotica /dispar* 9. *S. stercolaris* 10. *C. parvum*   11. I . beli  12. others ( specify*)---------------------*  13 No ova-parasite seen |
| Q49 | Type of infection | 1. Single parasitism  2. Double infection  3. Triple infection |
| Q50 | Household waste disposal | 1. Buried/Burned 2. Open field 3. Used as fertilizers |
